# Use of biomimetic hexagonal surface texture in friction against lubricated skin

**DOI:** 10.1098/rsif.2014.0113

**Published:** 2014-05-06

**Authors:** Alexey Tsipenyuk, Michael Varenberg

**Affiliations:** Department of Mechanical Engineering, Technion, Haifa 32000, Israel

**Keywords:** biomimetics, tribology, surface patterning, sliding

## Abstract

Smooth contact pads that evolved in insects, amphibians and mammals to enhance the attachment abilities of the animals' feet are often dressed with surface micropatterns of different shapes that act in the presence of a fluid secretion. One of the most striking surface patterns observed in contact pads of these animals is based on a hexagonal texture, which is recognized as a friction-oriented feature capable of suppressing both stick–slip and hydroplaning while enabling friction tuning. Here, we compare this design of natural friction surfaces to textures developed for working in similar conditions in disposable safety razors. When slid against lubricated human skin, the hexagonal surface texture is capable of generating about twice the friction of its technical competitors, which is related to it being much more effective at channelling of the lubricant fluid out of the contact zone. The draining channel shape and contact area fraction are found to be the most important geometrical parameters governing the fluid drainage rate.

## 1. Introduction

During the last decade, temporary attachment systems of terrestrial animals have become the focus of interdisciplinary scientific research aiming at revealing and possibly using functional principles underlying their amazing performance [1,2]. These systems are based on two equally important types of attachment pads relying on hairy or smooth architectures [3], which have received, however, different degrees of attention. Hairy attachment systems have spurred much research that resulted in the appearance of a whole new direction dealing with the so-called ‘gecko adhesion’ effect with more than 500 papers devoted to the subject since 2000 [4]. Smooth attachment systems, on the other hand, have received far less attention, which, given their no less intriguing properties, for instance, high resistance to sliding [5–8], calls for further investigation.

**Figure 1.**
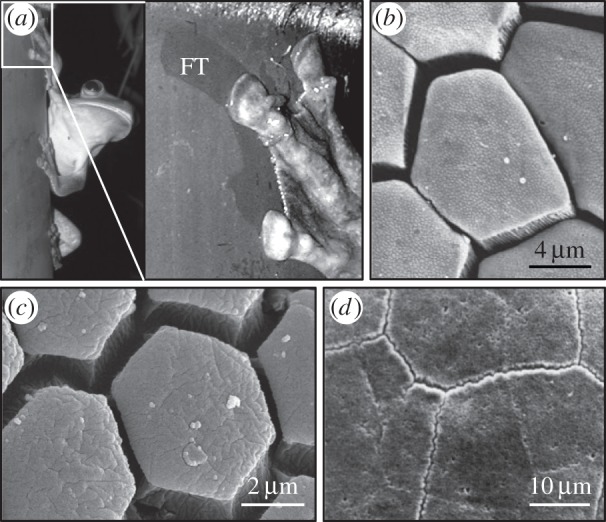
(*a*) Rusty tree frog, *Hypsiboas boans*, on a tree trunk (L. Allan 2013, personal communication). FT, fluid secretion trace left on the tree surface owing to the limb sliding before friction stopped the motion. (*b–d*) Surface pattern in attachment pads of White's tree frog, *Litoria caerulea* [17], great green bush cricket, *Tettigonia viridissima* [9], and mushroom-tongued salamander, *Bolitoglossa odonelli* [18], respectively.

Focusing on smooth contact pads evolved in insects, amphibians and mammals to enhance the attachment abilities of the animals’ feet [9–11], we see that they are often dressed with different surface micropatterns [12–15] acting in the presence of a fluid secretion, for example an oil-in-water emulsion in insects [16]. Moreover, some of the animals possessing smooth patterned lubricated pads exhibit jumping behaviour, which requires high friction in both pushing off and landing (figure 1*a*; L. Allan 2013, personal communication).

One of the most striking surface textures observed in contact pads of these animals is based on a hexagonal pattern, which was evolved independently in representatives of bush crickets [9], tree and torrent frogs [17] and mushroom-tongued salamanders [18] (figure 1*b–d*). Mimicked using soft elastomers and tested in both dry and wet conditions, the hexagonal surface pattern was recognized as a friction-oriented feature capable of suppressing both stick–slip and hydroplaning while enabling friction tuning [19–21]. Having this biomimetic hexagonal surface texture at hand, we were keen to answer the question of how it compares to advanced technical surfaces developed for use in similar conditions. This required finding a suitable well-engineered surface, which was detected in disposable safety razors.

Modern disposable safety razors consist of three units fixed to the common base and used for (i) stretching the skin, (ii) shaving the hair, and (iii) relieving any discomfort felt during shaving. The skin-stretching unit is intended to replace the hand that was once used for this purpose while scraping at the skin with the razor in the other hand. This unit is composed of an array of high-aspect-ratio (height to width) flexible round-ended projections located ahead of the blades and works through friction. The inherent problem of this unit is related to the presence of shaving lubricant, which is needed for more comfortable hair cutting. The lubricant separates the skin and the skin stretcher, thus decreasing the useful friction. This difficulty is very closely related to the problem of hydroplaning solved in evolution of smooth attachment systems. Thus, the skin-stretching unit in modern safety razors seems to present an ideal example of a technical element where the biomimetic hexagonal surface texture can be tested.

## 2. Material and methods

### 2.1. Specimens, preparations and conditions

**Figure 2.**
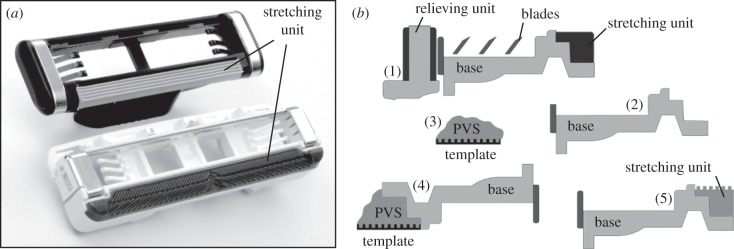
(*a*) Disposable safety razor cartridges of two leading manufacturers with skin-stretching units fixed to their bases while blades and skin-relieving units are removed. (*b*) Schematic of the specimen preparation procedure. (1) Section view of a commercial safety razor cartridge. (2) Safety razor base with stretching, shaving and relieving units removed. (3) Polymerizing PVS poured onto the negative template. (4) Safety razor base pressed against polymerizing PVS laying on the negative template. (5) Safety razor base with the PVS skin-stretching unit fixed and cut to size.

Patterned surfaces (table 1) were produced by pouring two-compound polymerizing polyvinylsiloxane (PVS; Coltène Whaledent AG, Altstätten, Switzerland) into negative templates prepared by different techniques. To fabricate moulding templates for small scale hexagons, we used photolithographic patterning of an SU-8 photoresist on a Si wafer [22]. Three-dimensional printing of hexagonally shaped walls on a smooth glass substrate was used to produce a moulding template for large-scale hexagons. To prepare negative templates of commercial skin stretchers, we first removed the blades and skin-relieving units from original cartridges (figure 2*a*), and then used these cartridges as patterns for fabrication of templates [23], which were made using G-8475 chemically inert polyurethane elastomer (Polyurethane Ltd, Haifa, Israel). Immediately after pouring into a template, the polymerizing PVS was covered with the safety razor base (with all original units removed) and pressed to squeeze superfluous polymer out of the gap. After polymerization, the PVS cast (Young's modulus of about 3 MPa [24]) fixed to the safety razor base was cut to the size of the device (figure 2*b*). The tests were performed with the cartridges slid against the skin of a 30-year-old male volunteer (one of the authors) on an inner part of an intact forearm skin lubricated by Gillette shaving foam (Procter & Gamble, Boston, MA, USA). The temperature of the forearm skin was 32°C. The temperature and relative humidity in the laboratory were 24°C and 45%, respectively.

**Table 1.** Surface textures used in this study.

surface texture	projection contact shape	projections profile	projection base thickness/diameter (μm)	projection height (μm)	centre-to-centre distance (μm)
C1	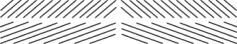	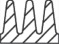	280	380	380
C2	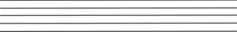	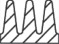	300	510	500
B1	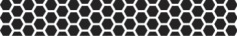	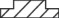	610	460	1212
B2	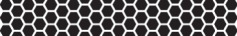	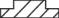	50	25	79
B3	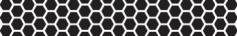	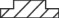	50	40	79
B4	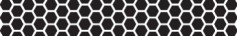	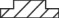	50	40	52
R	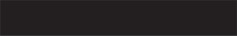	reference smooth surface (*R*_a_ = 0.04 μm) replicated from a microscope slide

### 2.2. Equipment

**Figure 3.**
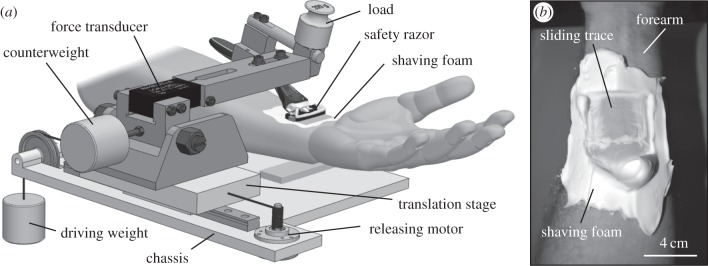
(*a*) Schematic of the experimental set-up and (*b*) sliding trace left on a forearm lubricated by shaving foam.

Surface appearance of the specimens used was inspected with a Leica M125 optical stereomicroscope (Leica GmbH, Wetzlar, Germany) and imaged in a FEI Quanta 200 environmental scanning electron microscope (FEI Co., Brno, Czech Republic). The tests were performed on a home-made tribometer (figure 3*a*) that incorporates two units used for driving and measuring purposes. The drive unit consists of a translation stage mounted on a linear bearing and drawn by a dead weight, which is released by an electrical motor at a constant controlled velocity. The measurement unit, which was assembled on the driving unit for these tests, consists of a 50 N load cell Z8 (spring constant *k* = 380 N m^−1^, HBM, Darmstadt, Germany) mounted on a hinged balanced arm, which allows loading the contact with known weights and determining the friction force arising due to the motion of the safety razor relative to the skin. The measurements were sampled with a multifunctional data acquisition board Lab-PC-1200 (National Instruments Co., Austin, TX, USA) and processed using a LabVIEW software package (National Instruments Co.).

### 2.3. Procedure

Before each experiment, the specimens and the forearm were washed with de-ionized water. Each test started by spreading an even few-millimetre-thick layer of shaving foam on the forearm and bringing the safety razor into contact with the skin (always at the same point). After applying (and maintaining) a normal load of 2 N, which was determined to be similar to the one acting in real shaving, the translation stage was moved at a velocity of 3 mm s^−1^ for a distance of 25–30 mm (figure 3*b*), while the tangential force resisting the skin stretcher motion was recorded. After each trial, the forearm was washed until the skin no longer felt soapy. Then, without drying the skin, a new layer of shaving foam was applied. At least 15 repetitions of each test type were made. After finishing all trials of each test type, the experiments were postponed to at least the next day, allowing the skin to recover. No redness or other skin disorders were observed.

## 3. Results and discussion

**Figure 4.**
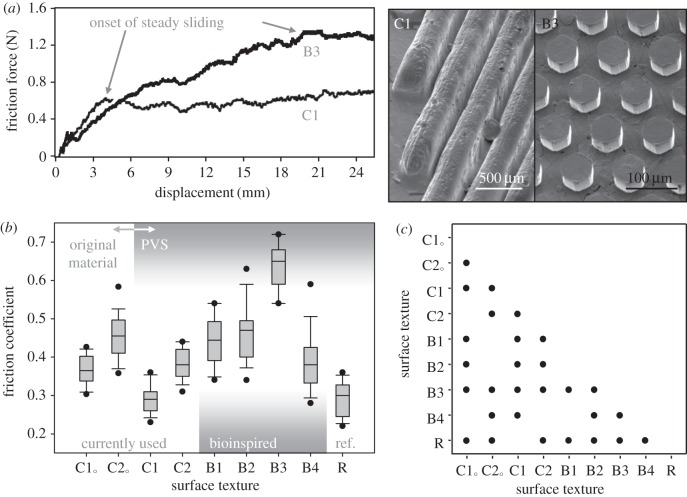
Friction obtained on human skin with real safety razor cartridges having blades and skin-relieving units removed and equipped with skin-stretching units dressed with original and biomimetic surface textures. (*a*) Characteristic friction curves as a function of displacement for C1 and B3 surface textures (shown in inset). (*b*) Complete dataset of kinetic friction coefficients obtained at the end of the sliding stroke. The data are presented using box-and-whisker diagrams, where the band inside the box is the median, the bottom and top of the box are the 25th and 75th percentiles, the ends of the whiskers are the 10th and 90th percentiles and the dots are the 5th and 95th percentiles. (*c*) Statistical differences in the mean kinetic friction coefficient values among different surface textures (one-way ANOVA, all pairwise multiple comparison Holm–Sidak procedure, *p* < 0.05). Symbols indicate significant difference.

Figure 4 presents friction data obtained on human skin with real disposable safety razor cartridges having blades and skin-relieving units removed and equipped with different skin-stretching units dressed with both original and biomimetic surface textures (see Material and methods section for details). To get a preliminary idea as to whether bioinspired surfaces can compete with commercial rivals, we have arbitrarily chosen several different characteristic sizes of hexagonal pattern, which are summarized in table 1 along with those of commercial skin stretchers. For correct comparison, all stretching units were cast from the same PVS elastomer.

Characteristic friction curves are shown as a function of displacement in figure 4*a*. Current commercial skin stretcher demonstrates typical sliding behaviour when, after a short transient is completed at about 4 mm displacement, the stretcher starts moving relative to the skin with nearly constant friction force. This means that sliding conditions do not change and the amount of lubricant entering the contact equals the amount being drained, while a certain constant volume of lubricant is trapped between the surfaces. The biomimetic texture created on the same stretcher demonstrates similar behaviour but different characteristic values. The transient period, during which the lubricant initially trapped in the contact escapes gradually from the interface through the net of adjacent interconnected channels [19], is much longer, resulting in a higher friction when the steady sliding is eventually reached. This means that a much smaller amount of lubricant remains trapped between the sliding surfaces in the second case. Thus, the biomimetic texture based on flat-end low-aspect-ratio protrusions (B3 inset in figure 4*a*) seems to outperform its current commercial rival based on a high-aspect-ratio wiper-like geometry (C1 inset in figure 4*a*), providing much more effective resistance to the lubricating action of the shaving fluid.

Complete dataset of kinetic friction coefficients obtained at the end of the sliding stroke is shown in figure 4*b*, where friction of the commercial stretching units made of the original materials (unspecified thermoplastic elastomers) is also given for reference. Comparing the performance of the textures created on the surface of the same PVS elastomeric material (figure 4*c*), we come to the following conclusion. The surface textures developed by the two leading safety razor manufacturers (marked as C1 and C2), which represent a well-engineered example of the current commercial technology, demonstrate friction that is the lowest in the case of texture C1, whose performance is indistinguishable from that of reference smooth surface R, or, in the case of texture C2, is comparable to the worst result demonstrated by biomimetic texture B4. Biomimetic texture B1 consisting of hexagons of 610 μm in diameter performs better than commercial textures C1, C2 and B4, similar to texture B2 and less effective than texture B3 measuring the highest friction. Bearing in mind that textures B2, B3 and B4 are built by hexagons of 50 μm in diameter packed with different densities, this means that the number of contact projections alone, ranging from 125 in B1 to about 30 000 in B3 and 69 000 in B4, cannot be the parameter determining the differences in performance of the biomimetic textures tested. Analysing the behaviour of textures B1, B2, B3 and B4, which consist of hexagons of different diameters, heights and centre-to-centre distances, we see that neither area density nor aspect ratio (table 2) usually used for characterization of surface texture performance [25,26] can explain the observed results either, which calls for finding another parameter that can satisfy the data.

**Table 2.** Analysis of bioinspired texture geometry (see the electronic supplementary material for details).

surface texture	mean kinetic friction coefficient	area density^a^	aspect ratio^b^	channel section shape factor^c^	contact area use factor^d^	efficiency factor^e^
B1	0.441	0.19	0.75	0.67	0.24	0.16
B2	0.460	0.30	0.50	0.70	0.43	0.30
B3	0.639	0.30	0.80	0.89	0.43	0.38
B4	0.389	0.70	0.80	0.21	0.43	0.09

^a^Ratio of projection contact area to total stretcher area.

^b^Ratio of projection height to projection diameter.

^c^Ratio of *min*(channel width, channel depth) to *max*(channel width, channel depth).

^d^Ratio of *min*(contact area, non-contact area) to *max*(contact area, non-contact area).

^e^Product of channel section shape factor by contact area use factor.

Coming back to the problem definition, we recall that in order to achieve high friction, the surface has to have as least as possible lubricant in as large as possible contact, which can be obtained by optimizing the efficiency of a drainage system. This efficiency is formed by (1) the projection ability to (a) squeeze the lubricant to adjacent channels and (b) form good contact with the mating surface and (2) the channel ability to (a) receive the lubricant and (b) remove the lubricant out of the interface. This is a complex problem dealing with the effects of spatially and temporally changing thickness of lubricant layer and deformation of solid surfaces [27], whose solution is beyond the scope of this paper. Trying to analyse this problem qualitatively, we can, however, come up with a simple parameter capable of explaining the observed results to a first approximation.

First of all, both wide shallow and narrow deep channels will be less effective in lubricant drainage than a channel of equal width and depth owing to their larger hydraulic resistance [28]. This allows us to define the channel section shape factor (table 2; electronic supplementary material) as the ratio of *min*(width, depth) to *max*(width, depth), which increases with the channel drainage efficiency. Second, it is clear that both (1) the texture having large contact area and small non-contact area (area of channels) and (2) the texture having small contact area and large non-contact area will be less effective than the texture having comparable contact and non-contact areas. This follows from that, in case (1), it will be difficult to squeeze the lubricant from large contact to narrow channels and, in case (2), the lubricant will be easily squeezed out, but the contact area and, hence, friction will be small. This allows us to define the contact area use factor as the ratio of *min*(contact area, non-contact area) to *max*(contact area, non-contact area), which also increases with the system drainage efficiency. It is worth mentioning that, in agreement with an optimum of contact area use factor, more or less equal ratio of contact to non-contact area is observed in pads of tree frogs sitting on a substrate [5]. Interestingly, the product of channel section shape factor by contact area use factor allows ranking correctly the biomimetic textures with respect to their frictional performance (table 2). This result, however, has to be treated with caution due to its approximated character and serve as a guide showing possible directions for further exploration using both experimental and theoretical approaches.

**Figure 5.**
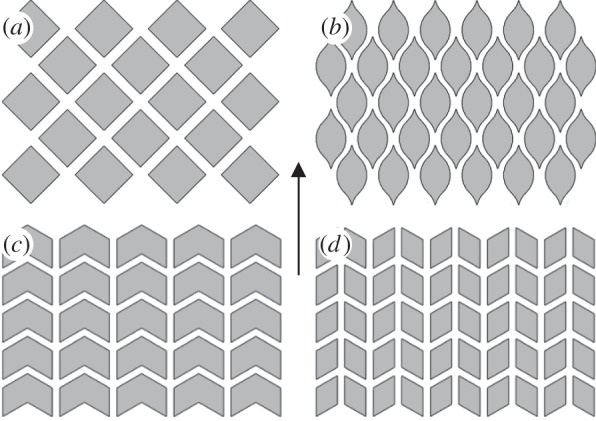
(*a*–*d*) Alternative configurations of bioinspired surface textures based on flat-ended protrusions that may also be efficient in fluid drainage from the unidirectionally sliding lubricated contact. Arrow shows the direction of sliding.

The contact area use and the draining channel shape are, however, not the only parameters defining the efficiency of the system, as the channel orientation may also affect the fluid escape rate when the direction of sliding motion is clearly defined (as in shaving). This can be demonstrated by the examples seen again in nature, when, for instance, looking at the differences in the toe pad cells evolved in tree and torrent frogs. It is found that more straightened and specifically oriented channels in between the contact cells in torrent frogs, which live near waterfalls in high flow conditions, can facilitate drainage of excess fluid underneath the pad [29,30]. Taking this into account, we can suggest several alternative geometries of flat contact protrusions that may also be efficient in fluid drainage from the unidirectionally sliding lubricated contact (figure 5). The patterns shown in figure 5*a–c* represent an abstraction of the geometry of single-contact cells observed in torrent frogs [29,30], and the pattern shown in figure 5*d* represents further development of the pattern from figure 5*c*. Tribological efficiency of these textures, however, requires further verification.

Returning to the analysis of the data presented in figure 4*b*, we can add that even though the PVS elastomer is not the best material for friction-based skin stretcher, as becomes evident from the comparison of original and PVS-based commercial textures (in both C1 and C2 cases), the biomimetic surface texture generates significantly higher friction than its commercial competitors, even when made from a non-optimal material. Interestingly, the problem of low friction indeed concerns the safety razor manufacturers, as can be seen from the gradual changes we observe in their razors. These changes involve increasing the size of the skin stretcher when increasing the number of blades, and adding special elements intended to facilitate removal of excessive lubricant from the interface. It is also worth noting here that according to the volunteer report, no stick–slip events or any other discomfort were experienced and the biomimetic texture felt much softer in touch than its commercial rivals, which may be related to a more even distribution of the contact load. Thus, the reported results open wide perspectives in improving the performance of modern disposable safety razors and may become yet another example of the successful application of bioinspired technology in our daily life.

## 4. Conclusion

Our experimental results suggest that friction of biomimetic hexagonal surface texture tested against lubricated skin depends on the efficiency of the draining net channelling fluid out of the interface and the size of the contact built by the surface projections. Applied in real safety razors for friction-based stretching of lubricated skin, the biomimetic surface designed to facilitate drainage of excessive fluid from the contact zone is capable of generating about twice the friction of commercial surfaces in current use.
